# Complete mitochondrial genome of the silkworm strain, Chilseongjam *Bombyx mori* (*Lepidoptera*: *Bombycidae*), with a unique larval body marking

**DOI:** 10.1080/23802359.2019.1660278

**Published:** 2019-09-17

**Authors:** Seong-Wan Kim, Min Jee Kim, Kee-Young Kim, Seong-Ryul Kim, Iksoo Kim

**Affiliations:** a Department of Agricultural Biology, National Academy of Agricultural Science, Rural Development Administration, Wanju Gun, Republic of Korea;; b Department of Applied Biology, College of Agriculture and Life Sciences, Chonnam National University, Gwangju, Republic of Korea

**Keywords:** Mitochondrial genome, *Bombyx mori*, silkworm strain, Chilseongjam, phylogeny

## Abstract

Recently, a new silkworm strain with a peculiar larval marking and rare cocoon colour was bred in Korea for educational learning and exhibition. In order to obtain the genetic information of the newly bred strain, Chilseongjam *Bombyx mori* (Lepidoptera: Bombycidae), its complete mitochondrial genome (mitogenome) was sequenced. The mitogenome is 15,660 bp in length, contains a typical set of genes, and has gene arrangement and composition typical of Lepidoptera. However, the Chilseongjam strain mitogenome is 4–36 bp longer than 19 other strains originating from other countries and 16 bp shorter than the whole genome of a Korean Hukpyobeom strain. In particular, the Chilseongjam strain has an intergenic spacer sequence that is shorter than that of the Hukpyobeom strain at the tRNA^His^ and ND4 junction as it has fewer microsatellite-like AT repeats. Phylogenetic analyses conducted using a total of 21 silkworm strains originating from nine countries revealed a few subgroups with moderate-to-high nodal support (80–94%). The Korean Chilseongjam strain formed a relatively strong subgroup (85%) with a Japanese strain (J106) instead of the Korean Hukpyobeom strain.

The domestic silkworm, *Bombyx mori* (Lepidoptera: Bombycidae), has long been bred for the production of targeted strains. In Europe and Asia, more than 3000 different silkworm strains are maintained with diverse characteristics (Nagaraju 2000; Ryu et al. 2003). In Korea, approximately 300 silkworm strains are registered as national genetic resources and are cultured annually. In 2016, a silkworm strain with a peculiar outward appearance and rare cocoon colour (light green) was bred for educational learning and exhibition in Korea (Kim et al. 2018). The strain has a pair of peculiar larval markers with a dark brown/black grey colour on seven segments of its dorsal abdomen and has light green cocoon colour (Figure 1). Therefore, a small but significant amount of genetic information on the strain is essential as a national genetic resource. We sequenced the complete mitochondrial genome (mitogenome) of the strain, which may provide enough genetic information for comparison with pre-existing global strains.

**Figure 1.**
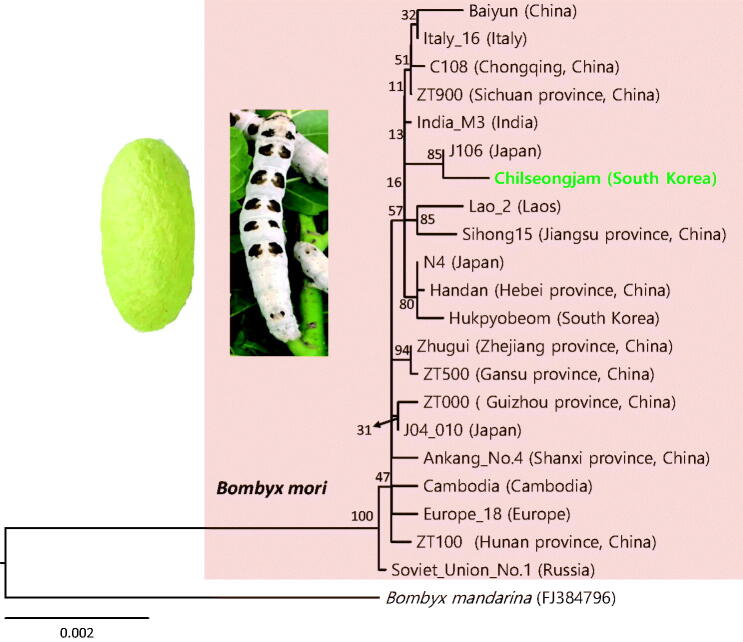
Phylogenetic tree of *Bombyx mori*. The maximum-likelihood (ML) method was used for phylogenetic analysis based on the concatenated sequences of 13 PCGs and 2 rRNAs. The numbers at each node specify the bootstrap percentages of 1000 pseudoreplicates. The scale bar indicates the number of substitutions per site. The wild silkworm (*Bombyx mandarina,* FJ384796, Hu et al. 2010) was utilized as the outgroup. The GenBank accession numbers are as follows: Europe_18, GU966607 (Li et al. 2010); ZT500, GU966611 (Li et al. 2010); Zhugui, GU966609 (Li et al. 2010); ZT000, GU966613 (Li et al. 2010); J04_010, GU966612 (Li et al. 2010); Baiyun, KM279431 (Zhang et al. 2016); Italy_16, GU966596 (Li et al. 2010); C108, GU966630 (Li et al. 2010); ZT900, GU966600 (Li et al. 2010); India_M3, GU966595 (Li et al. 2010); Lao_2, GU966610 (Li et al. 2010); Sihong15, GU966617 (Li et al. 2010); Handan, GU966628 (Li et al. 2010); J106, GU966615 (Li et al. 2010); Hukpyobeom, MK613835 (Kim et al. 2019); N4 GU966602 (Li et al. 2010); Ankang_No.4, GU966614 (Li et al. 2010); Cambodia, GU966601 (Li et al. 2010); ZT100, GU966603 (Li et al. 2010); and Soviet_Union_No.1, GU966599 (Li et al. 2010).

One larva of the Chilseongjam *B. mori* strain (Jam 321) was collected from the annual culturing samples of the government institute in Korea and subsequently deposited at the Chonnam National University, Korea, under accession no. CNU8290. To sequence its mitogenome, whole genome sequencing was performed on the NextSeq-500 platform (Illumina, San Diego, CA). The genome was constructed by *de novo* assembly using a GenBank-registered silkworm mitogenome (NC_002355; Unpublished). Due to certainty in the final genome sequence, no Sanger-based gap filling was conducted. The genomic sequences were compared to 20 silkworm strains originating from nine countries (China, Japan, Korea, India, Laos, Europe, Italy, Soviet Union, and Cambodia), including one Korean Hukpyobeom strain (Li et al. 2010; Zhang et al. 2016; Kim et al. 2019).

The complete 15,660 bp Chilseongjam mitogenome is composed of typical gene sets (two rRNAs, 22 tRNAs, and 13 protein-coding genes (PCGs)) and a major non-coding A + T-rich region (GenBank acc. no. MN103530). The Chilseongjam mitogenome has a CGA codon as the start codon for COI, found almost universally in Lepidoptera and has gene arrangement identical to that of the ditrysian Lepidoptera (Kim et al. 2010; Park et al. 2016). The Chilseongjam mitogenome was 4–36 bp longer than the 19 strains originating from other countries (Li et al. 2010; Zhang et al. 2016), but 16 bp shorter than the whole genome of a Korean Hukpyobeom strain (Kim et al. 2019). In particular, the Chilseongjam strain had an intergenic spacer sequence 18 bp shorter than that of Hukpyobeom (54 bp *vs*. 72 bp) as it had fewer microsatellite-like AT repeats at the tRNA^His^ and ND4 junction.

For phylogenetic analysis, 13 PCGs and two rRNA genes were aligned, along with the outgroup species *Bombyx* *mandarina*. The maximum likelihood method was conducted using RAxML-HPC2 version 8.0.24 (Stamatakis 2014) in CIPRES Portal version 3.1 (Miller et al. 2010). Phylogenetic analyses conducted using a total of 21 silkworm strains revealed that global silkworm strains formed one large monophyletic group with the highest nodal support, but a few subgroups were supported by moderate-to-high nodal support (80–94%). The Korean Chilseongjam strain formed a relatively strong subgroup (85%) with a Japanese strain (J106) instead of the Korean Hukpyobeom strain. We hope that the Chilseongjam mitogenome sequence is utilized for the subsequent development of strain-diagnostic markers.
